# A DNA-based pattern classifier with *in vitro* learning and associative recall for genomic characterization and biosensing without explicit sequence knowledge

**DOI:** 10.1186/1754-1611-8-25

**Published:** 2014-11-06

**Authors:** Ju Seok Lee, Junghuei Chen, Russell Deaton, Jin-Woo Kim

**Affiliations:** Bio/Nano Technology Laboratory, Institute for Nanoscience and Engineering, University of Arkansas, Fayetteville, Arkansas 72701 USA; Department of Biological and Agricultural Engineering, University of Arkansas, Fayetteville, Arkansas 72701 USA; Cell and Molecular Biology Graduate Program, University of Arkansas, Fayetteville, Arkansas 72701 USA; Department of Chemistry and Biochemistry, University of Delaware, Newark, Delaware 19716 USA; Department of Electrical and Computer Engineering, University of Memphis, Memphis, Tennessee 38117 USA; Department of Chemistry, Seoul National University, Seoul, Republic of Korea

**Keywords:** Biological memory protocol, *In vitro* learning and recall, Microarray, Genomic status, Ecological and environmental monitoring, Biological and biomedical sensing

## Abstract

**Background:**

Genetic material extracted from *in situ* microbial communities has high promise as an indicator of biological system status. However, the challenge is to access genomic information from all organisms at the population or community scale to monitor the biosystem’s state. Hence, there is a need for a better diagnostic tool that provides a holistic view of a biosystem’s genomic status. Here, we introduce an *in vitro* methodology for genomic pattern classification of biological samples that taps large amounts of genetic information from all genes present and uses that information to detect changes in genomic patterns and classify them.

**Results:**

We developed a biosensing protocol, termed Biological Memory, that has *in vitro* computational capabilities to “learn” and “store” genomic sequence information directly from genomic samples without knowledge of their explicit sequences, and that discovers differences *in vitro* between previously unknown inputs and learned memory molecules. The Memory protocol was designed and optimized based upon (1) common *in vitro* recombinant DNA operations using 20-base random probes, including polymerization, nuclease digestion, and magnetic bead separation, to capture a snapshot of the genomic state of a biological sample as a DNA memory and (2) the thermal stability of DNA duplexes between new input and the memory to detect similarities and differences. For efficient read out, a microarray was used as an output method. When the microarray-based Memory protocol was implemented to test its capability and sensitivity using genomic DNA from two model bacterial strains, *i.e., Escherichia coli* K12 and *Bacillus subtilis*, results indicate that the Memory protocol can “learn” input DNA, “recall” similar DNA, differentiate between dissimilar DNA, and detect relatively small concentration differences in samples.

**Conclusions:**

This study demonstrated not only the *in vitro* information processing capabilities of DNA, but also its promise as a genomic pattern classifier that could access information from all organisms in a biological system without explicit genomic information. The Memory protocol has high potential for many applications, including *in situ* biomonitoring of ecosystems, screening for diseases, biosensing of pathological features in water and food supplies, and non-biological information processing of memory devices, among many.

**Electronic supplementary material:**

The online version of this article (doi:10.1186/1754-1611-8-25) contains supplementary material, which is available to authorized users.

## Introduction

Nucleic acid technology has become an indispensible tool in medical diagnosis, microbial ecology, environmental microbiology, *etc.* by providing specific, sensitive detection of genes in chemically and biologically complex backgrounds. However, genome-enabled studies have focused on specific individual organisms. Conventional techniques (*i.e.,* polymerase chain reaction (PCR)-based and gel-based methods) for studying DNA samples require prior knowledge of the sequence, either for PCR primers or for attachment to a target DNA. By focusing on known genes, information from other unknown genes is lost. Multiple genes or multiple infectious microorganisms are known to be involved in many human diseases; however, only a fraction of these genes has been identified, even after the sequencing of the human genome and several microorganisms. The functions of many proteins encoded by these genes are unknown [1]. In addition, studies have estimated that there are approximately 4 × 10^3^ to 10^4^ microbial species per gram of soil, but only less than 1% of microorganisms in nature are observable with the standard culturing techniques [2]. These in turn generated a renewed demand for innovative approaches that can quickly, exhaustively, and intelligently detect and classify gene expression profiles. It is possible to extract genomic samples, such as DNA and RNA, from any biological sample, such as soil, water, and biological specimens, without knowing the genomic identities of the samples. The complementary DNA (cDNA) synthesis from messenger RNA (mRNA) is also well established, either using oligo-dT or random primers. Recent advances in the next-generation sequencing (NGS) technology opened the possibility of *de novo* sequencing without any pre-existing genomic references [3]. NGS can generate very large volumes of short sequencing reads of genomic DNA (gDNA) at markedly reduced prices and faster rates, and the massive data can be reassembled *de novo*. However, substantial challenges exist for the *de novo* applications of NGS, including high error rates, massive information technology systems for data processing and storage, *etc.*; hence, NGS with *de novo* assembly is still limited to species-specific applications, including bacterial genomes and mammalian bacterial artificial chromosomes [3–8]. Thus, a challenge is to discover more efficient and effective ways to tap the large amounts of genetic information from a biological sample (*i.e.,* gDNA) or all expressed genes in the biological sample (*i.e.,* cDNA from mRNA), and to use that information to detect changes in genomic patterns and classify them.

**Figure 1:**
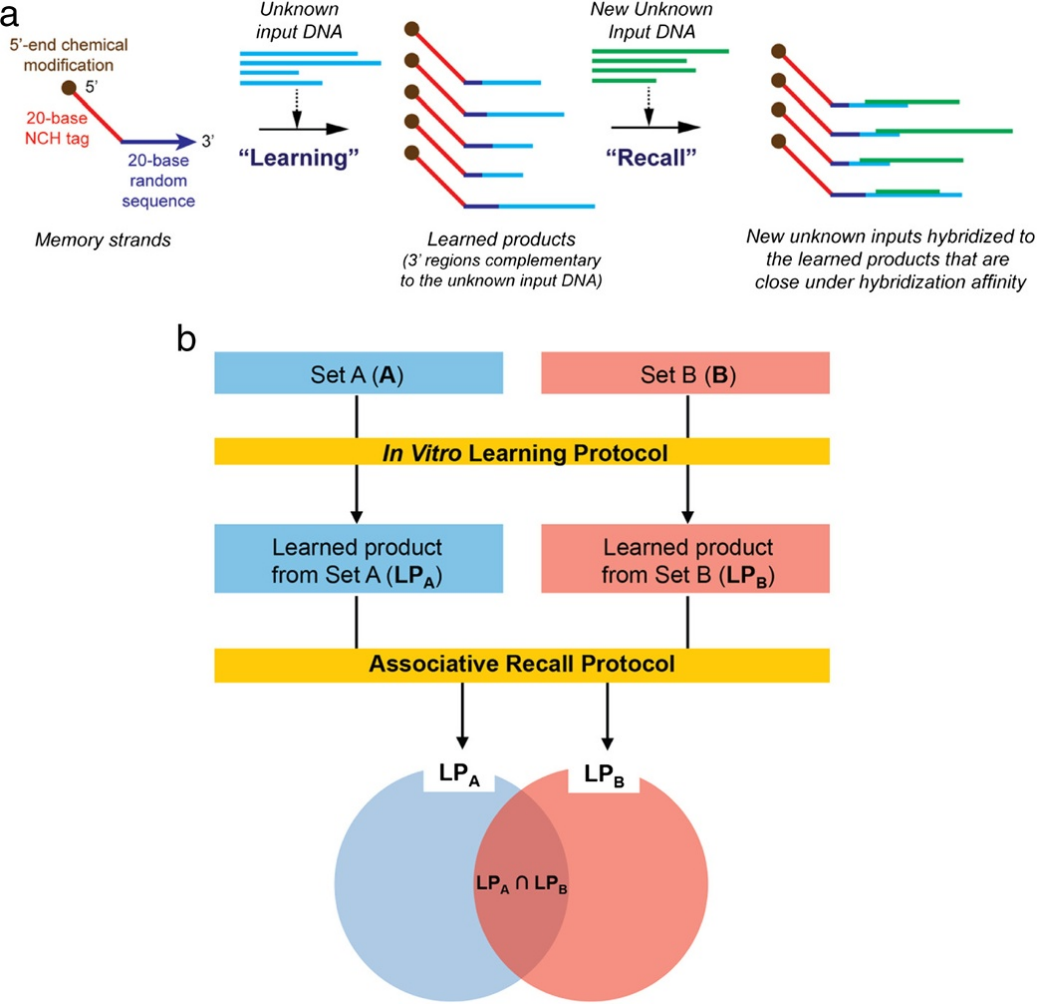
**Schematic and principle of a Biological Memory: (a) Schematic of a Biological Memory with** ***in vitro*** **learning and associative recall and (b) principle of Biological Memory using two sets of genomic populations.** The learned product of set A (LP_A_) is a subset of set A, and LP_B_ is a subset of set B. Theoretically, LP_A_ (or LP_B_) is same to set A (or set B). The [LP_A_ – LP_B_] represents nothing but set A, and the [LP_B_ – LP_A_] does set B. Hence, set A and set B can be discriminated through DNA hybridizations between LP_A_ and LP_B_ and their comparisons.

To this end, we introduce a new *in vitro* methodology for genomic pattern classification of biological samples. We designed and implemented a biosensing protocol, termed Biological Memory, that has *in vitro* computational capabilities to learn genomic sequence information from genomic samples, and that can detect changes in the genomic information from biological samples without explicit knowledge of their genomic sequence or composition (Figure 1). The idea of processing large amounts of information in a test tube, not on a conventional solid-state computer, presents the possibility of working with gDNA or cDNA on a community or population scale. The Biological Memory does not seek to determine the complete sequence information from biological samples. The Biological Memory is a laboratory protocol that, through DNA hybridization reactions with random probes, matches sequence patterns and stores the DNA sequence information in a DNA-based memory *in vitro*. Then, it matches or “recalls” the stored information based upon sequence similarity to new input (*i.e.,* “associative recall”) [9]. Using a microarray as an output method, read out is easily accomplished and high-throughput. Multiplexed detection is possible. More conventional techniques would access the genomic information in the biological samples with known sequences, and then, rely upon a digital computer for processing the data. Similarly, the massive volumes of short sequencing reads of NGS must be processed digitally for their *de novo* assembly. Pattern recognition and interpretation of large amounts of gene expression or genomic data are difficult problems for conventional computers. However, the Memory does all its information processing *in vitro* in one massively parallel step and does not require sequencing, not only alleviating sources of errors associated with sequencing techniques and *in silico* data processing, but also allowing the classification of patterns from all organisms in biological samples at the population or community scale. This paper discusses the theoretical analysis of the microarray-based Biological Memory with DNA as a representative target and its experimental verification and optimization processes using gDNA from two model bacterial strains, *i.e., Escherichia coli* K12 and *Bacillus subtilis*, addressing basic understanding of the information processing capabilities of DNA, and the promises of the practical applications of those properties to biology and medicine.

### Design of Biological Memory with *in vitro*learning and associative recall

In the Biological Memory (Figure 1a), the initial sequences (termed memory tags) are a set of tag oligonucleotide sequences, to which random sequences are appended during their synthesis. The appended random sequences theoretically contain every possible sequence of a given length. The tag oligonucleotide sequences are designed to be independent of each other in that they do not hybridize to each other (*i.e.*, non-crosshybridizing [NCH]) [10–14] (termed NCH tag), and can be used for printing on a microarray slide for output or separating products (*e.g.,* magnetic bead separation) with proper chemical modifications (*i.e.,* amine, carboxyl, or biotin modifications). Also, the NCH tag could be utilized for other types of product separation or output techniques for downstream processing, such as DNA affinity column chromatography or DNA microarray with sequences complementary to the tag sequence attached to the column matrix or microarray slide. With simple and common recombinant DNA operations, such as polymerization, nuclease digestion, and DNA separation (*e.g.,* magnetic bead separation or column chromatography), the system learns the DNA sequences to which it is exposed. These learned sequences can then be stored as a DNA memory. Subsequently, the learned memory stands can be used to “recall” the input sequences, or sequences that are close under hybridization affinity.

**Figure 2:**
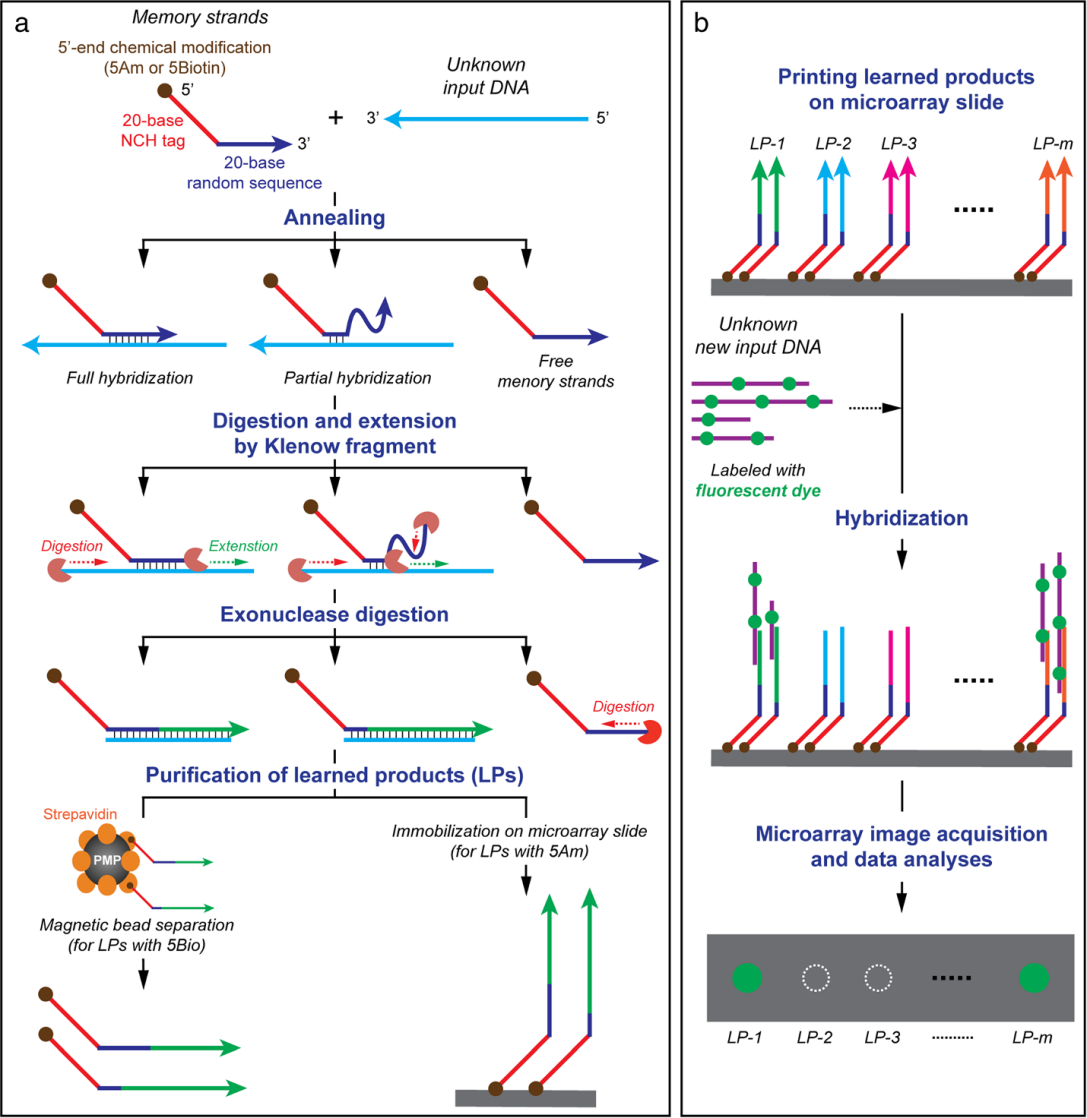
**Experimental Design of a Biological Memory: (a)** ***In vitro*** **learning protocol and (b) microarray-based associative recall protocol.**

Schematics of detailed protocols of the Memory designed for this study are shown in Figure 2. For learning (Figure 2a), initially, each memory tag consists of a 20-base NCH tag sequence with biotin- or amine-modified 5′ end (5Bio and 5Am, respectively), followed by a 20-base random probe sequences (R20). In this study, the 5′-end modifications are designed for the following downstream applications: (1) the 5′-end biotin-modified memory tags to separate the learned products using the streptavidin-coated magnetic beads, and (2) the 5′-end amine-modified memory tags for the immobilization of the learned products on to microarray slides. The 20-base NCH tag sequences in the memory tag are designed and experimentally confirmed, according to our established methods [10, 13] to be thermodynamically unfavorable at room temperature for hybridizing to any other DNA sequences except their exact complementary sequences. The input DNA (*e.g.,* gDNA extracted from a biological sample), which is to be learned, is mixed with the initial memory tags (*i.e.,* NCH tag plus R20 probe). The R20 probes anneal fully or partially at random locations on the input DNA. The subsequent single step polymerization by Klenow fragment not only digests the dangling end of the memory tags from the 3′ end until a double-stranded region is encountered, but also extends it by 5′ to 3′ polymerization to learn the input DNA. The free, unbound memory tags and input DNA are removed by exonuclease digestion. The extended memory strands (*i.e.,* tag plus extended Watson-Crick complement of input) are purified by commercially available magnetic bead separation. The products are single-stranded DNA with a unique tag attached to random length 3′ regions that are complementary to the input DNA, and that have undergone some amplification during polymerization. To learn additional inputs, the process is repeated with a different NCH tag. For recall (Figure 2b), unknown input is exposed to the learned memory strands. The input will hybridize to the learned memory sequences that are close to its Watson-Crick complement. In this study, the learned memory strands are printed on to a microarray slide for easy output. Its capability and sensitivity are evaluated using gDNA from two model bacterial strains, *i.e., E. coli* K12 and *B. subtilis*.

## Results and discussion

### Principle of the Biological Memory

An underlying hypothesis of the DNA-computing-inspired Biological Memory is that the products, which are learned *in vitro*, represent the entire DNA population in the input genomic sample (*i.e.,* gDNA or cDNA from a biological sample), and that the differences between input sets could be discriminated by separating the output hybridization patterns between the learned products. When it is assumed that there are two input populations (*e.g.,* set A and set B) (Figure 1b), through the *in vitro* learning protocol, the elements of each input population are stored in the learned products as content-addressable memory structures. Ideally, the learned products from the set A (LP_A_) and those from the set B (LP_B_) should contain every elements of set A and set B, respectively. The sets A and B can then be discriminated through DNA hybridization with their learned products using an appropriate detection system, such as microarray, as a part of the associative recall protocol. At a minimum, the Biological Memory should be able to distinguish between snapshots of a biological sample, from the environment or a living being.

One of the unique advantages of the Biological Memory stems from the utilization of random sequences in the initial memory tags. For example, the population of 20-base random sequences (R20) contains 4^20^ (~1.1 × 10^12^) different 20-base DNA sequences, which well exceed the number of 20-base segments in any genomes known so far (*e.g.,* ~3 × 10^9^ of human genome). Hence, it is postulated that, using the R20, the entire information of any genome could be captured and stored in a DNA-based memory, if the protocol is properly implemented. As the complexity of genomic samples increases, which would likely happen in real-world samples, for example from the environment or a living being, the Memory protocol could be easily adapted by using longer random sequences (*i.e.,* >R20) to capture more information in the complex samples. The storage procedure is called “learning” because the memory DNA acquires information from examples (*i.e.,* the input DNA), and does so without external knowledge of their genomic sequences. Also, incorporating longer random sequences, such as R20 or > R20, each memory tags can obtain high specificity even at ambient temperature as compared to the previously reported random primer method [15] with random oligonucleotides of 7 to 10 bases.

Furthermore, in the Biological Memory protocol, the genomic information is processed in one massively parallel step, just as searches have been done using DNA for solutions to hard computational problems [16]. Likewise, matching of stored patterns with new input is done in parallel, in one step. Similarity is implemented *in vitro* by degree of annealing between new input DNA and the learned memory sequences, thus providing a technique for recognizing patterns in different samples and detecting changes without requiring sequencing. By contrast, the established molecular biology techniques would acquire the genomic information through biological samples with known sequences, and then, depend on a conventional computer for processing the data. Even the new *de novo* NGS requires massive computer-based information processing for data assembly and their storage [3–8]. The Memory has *in vitro* capability to efficiently implement pattern recognition and interpretation of large amounts of genomic data that are difficult problems for conventional computers. The Memory is not a laboratory technique only to gather data for conventional computational analyses, but uses the massive scale of storage and parallelism of DNA as the computational tool to draw inferences on the entire *in vitro* knowledge base quickly and efficiently without any knowledge of sequences. This implies that the Memory can reason and extract knowledge in situations that involve both new and unknown information, which is hard to achieve with conventional laboratory techniques and computational analyses. Also, because there is no DNA sequencing, it is more effective and efficient by alleviating the inherent sources of errors, as well as costs associated with sequencing techniques. Moreover, DNA’s large storage capacity is used to store genomic information from a population or community in the sample for subsequent matching. The information is stored in a compact form, and can serve as a database of the status of the biological sample at a given moment in time. In other words, the Memory is capable of capturing global information on all organisms or whole genome gene expressions in biological samples under certain conditions, and recognizing patterns of contrast and commonality at the population or community level. When the learned memory sequences are attached to a DNA microarray, read out (*i.e.,* “recall”) is easily achieved and interpreted as either a positive or negative match. Also, the microarray’s high capacity for multiplexing is an added advantage, which other conventional gel- or PCR-based approaches cannot afford. Thus, a microarray would provide an ideal vehicle to implement the Biological Memory considering the enormous capacity of nucleic acids on each slide/chip. Finally, it should be noted that the microarray-based Biological Memory would also have significant utility as a simple, fast, flexible, and high-throughput non-gel/PCR based technical platform for genome studies, thus reducing the current reliance on conventional PCR and gel-based methodologies.

### Validation and characterization of the Biological Memory

An important property to characterize the Biological Memory is the ability of the learning and recall protocols to learn and differentiate different sets of DNA. In this study, the goal was to evaluate and verify the capabilities of the DNA-based Memory protocol, which include validating and optimizing learning of input DNA sequences using R20 and testing recall of the learned sequences, as well as its sensitivity using gDNA from two model bacterial strains, *i.e., E. coli* K12 and *B. subtilis*.

#### Development of *in vitro*learning protocol and its optimization

**Figure 3:**
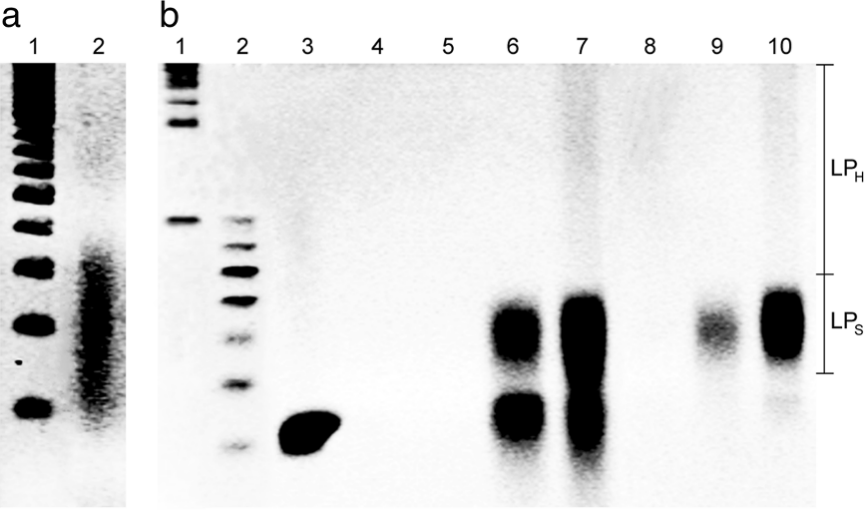
**Characterization of the learning protocol on the basis of the ethidium bromide staining denatured Urea polyacrylamide gel electrophoresis (Urea-PAGE) at 60°C. (a)** Digested *E. coli* gDNA with 4-12% Urea-PAGE. The lanes represent: (1) molecular size marker for single-stranded (ss) DNA of 100 to 1000 bases in 100-base increments and (2) digested *E. coli* gDNA (avg. ~200 bases). **(b)** Learning products with 4-20% Urea-PAGE. The lanes represent: (1) molecular size marker for ssDNA from 100 base in 100-base increments, (2) molecular size marker for ssDNA of 40 to 100 bases in 10-base increments, (3) memory tags (40 bases), (4) learning reaction mixture (*i.e.,* memory tag + digested gDNA) after annealing and digestion (A → D) (5) digested gDNA only after annealing and extension (A → E), (6) memory tags only after A → E, (7) learning reaction mixture after A → E, (8) digested gDNA only after annealing, extension, and digestion (A → E → D), (9) memory tags only after A → E → D, and (10) learning reaction mixture after A → E → D. The learning protocols were implemented with 5′-amine modified memory tags and the product of each run was separated by the magnetic bead separation. LP_H_ is the high-molecular weight (>80 bases) learned products and LP_S_ relatively short (50 – 80 bases) learned products.

To investigate the performance and efficiency of the *in vitro* learning protocol, the protocol was implemented at the annealing temperature of 25°C using enzymatically digested *E. coli* gDNA and a 5′-biotin labeled memory tag to separate out the learned products with the streptavidin-coated magnetic bead separation. Ethidium bromide staining denatured Urea polyacrylamide gel electrophoresis (Urea-PAGE) imaging revealed that an average length of the digested gDNA was around 200 bases (Figure 3a). Also, the Urea-PAGE gel imaging analyses indicated that, after implementing the learning protocol, the products were in general composed of 3 regions, *i.e.,* high-molecular weight learned products (LP_H_, >80 bases), relatively short learned products (LP_S_, 50 – 80 bases), and 40-base memory tag (Figure 3b), with varying concentrations depending upon the inputs and orders of reactions.

The learning protocol includes three key reaction steps (Figure 2a): the memory tag-input annealing (A), the strand extension by Klenow fragment (E), and *Exo* I digestion of unbound memory tags and inputs (D). After each step, the products are purified by the magnetic separation. When the orders of the reaction steps were compared, the results (Figure 3b) indicated that the order should be A → E → D. When digestion preceded extension, little or no extension occurred (Figure 3b, lane 4). However, after extension (Figure 3b, lane 7), followed by digestion (Figure 3b, lane 10), the amount of extended strands increased substantially and there existed much less unused memory tags compared to the initial tag concentration (Figure 3b, lane 3 vs lane 7). After digestion, most of the excess unbound memory tags were removed and only extended strands remained, whose molecular weights were higher than the memory tag (Figure 3b, lane 10). The length of the extended strands varied as indicated by the smear of DNA bands, but they were well within the range of the input gDNA length (Figure 3a, lane 2 vs. Figure 3b, lane 10). These results imply the successful learning of the input DNA sequences. For the rest of this study, all learning was performed on the basis of the A → E → D reaction sequence.

To further confirm the learning protocol, it was implemented with the following two negative controls, and the results were compared with the learned products with the positive control (*i.e.,* both input gDNA and memory tag): (1) memory tag only without input gDNA (Figure 3b, lanes 6 and 9) and (2) input gDNA only without memory tag (Figure 3b, lanes 5 and 8). The generalized annealing reactions in the learning protocol can be represented as: Input + Memory tag → Input-Input + Input-Memory tag + Memory tag-Memory tag. Among these, only the extended strands from Input-Memory tag complexes are the actual learned products, *i.e.* LP_I-M_. The other two, *i.e.,* learned products from the Input-Input (LP_I-I_) and Memory tag-Memory-tag (LP_M-M_), are by-products, which should be eliminated. The LP_I-I_ could be easily separated from the desired learned products (*i.e.,* LP_I-M_) due to the absence of a memory tag component in LP_I-I_, *i.e.,* no 5′-end modifications such as 5′ biotin. As expected, no products were observed after extension and magnetic separation (Figure 3b, lanes 5 and 8). Thus, the LP_I-I_ after annealing and extension should not affect the yield estimation of the final learned products, even though it could reduce the amount of available inputs for the memory tag annealing during the learning protocol and potentially reduce the final yield of the learned products. However, LP_M-M_ from the memory tag dimer could negatively affect the expected final outcomes, since it showed similar band pattern to the LP_I-M_ but does not have any information from the input. To investigate the possibility of the memory tag-memory tag duplex formation and their extension, the melting temperature distribution of various lengths of random oligonucleotides were calculated using OligoAnalyzer 3.1 under the reaction condition (1.6 μM of oligonucleotides; 10 mM Na^+^; 10 mM Mg^2+^; 4 mM dNTPs) (http://www.idtdna.com/analyzer/Applications/OligoAnalyzer/). The minimum estimated melting temperature of the R20 oligonucleotides under the learning reaction condition was around 42.6°C. This means that most of R20 could form a stable duplex at the reaction temperature (*i.e.,* 25°C). The memory tag concentration in the typical reaction volume in this study (50 μL) was 1.6 μM, which is equivalent to 4.8 × 10^16^ memory tag strands. The number of maximum independent sequences in the R20 is 1.1 × 10^12^ (*i.e.,* 4^20^) strands. Thus, the number of each unique R20 sequence in the reaction volume is approximately 4.3 × 10^4^. This indicates that the chances of self-hybridizations, especially perfect matches, between the Individual R20 themselves are extremely low (*i.e.,* 1 of 1.1 × 10^11^). However, there are still chances for partial hybridizations to form incomplete duplexes. According to the melting temperature estimation, around 7 bases could form a duplex under the reaction condition, since the estimated mean melting temperature of 7-base duplex was 25.7°C, and maximum melting temperature of 5-base duplex was 30.9°C, showing the possibility of the memory tag-memory tag annealing and extension through their partial hybridizations. The length of LP_M-M_ is depending on the location where hybridization occurs and estimated to be around 5 – 80 bases (see Additional file 1: Figure S1), as confirmed by the experimental result using only memory tags (Figure 3b, lanes 6 and 9). When comparing LP_I-M_ and LP_M-M_ (Figure 3b, lane 9 vs. 10), LP_H_ in LP_I-M_ not only had similar molecular weights as the inputs but also exceeded the possible length of LP_M-M_; thus, LP_H_ should be true learned products. However, the length of LP_S_ was around 50 to 80 bases, which overlaps with the estimated molecular weight distributions of LP_M-M_. Thus, LP_S_ of the learned products might be the mixture of LP_I-M_ and LP_M-M_. Nonetheless, it was noted that the concentration of LP_S_ in LP_I-M_ (Figure 3b, lane 10) increased substantially (>5 fold according to the gel intensity analysis) as compared to the negative control (Figure 3b, lane 9). This implies that LP_M-M_ might be present in the learned products, but its concentration would not be significant. The probability of memory tag-memory tag hybridization in the presence of input DNA should be lower than that with memory tag only; hence, the concentration of LP_M-M_ in the learned products should be lower than the memory tag only.

**Figure 4:**
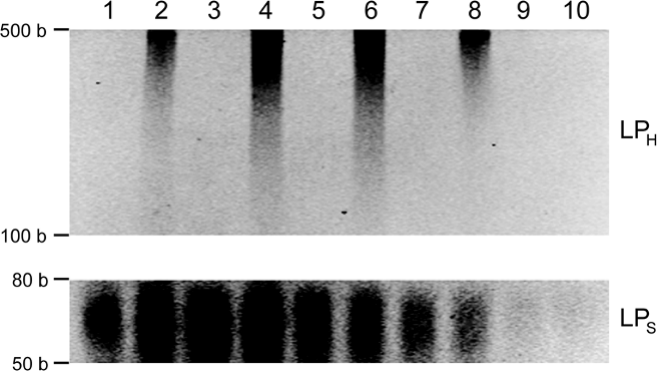
**Learning at various annealing temperatures for the protocol optimization: 25°C (lanes 1 and 2), 40°C (lanes 3 and 4), 55°C (lanes 5 and 6), 60°C (lanes 7 and 8), and 70°C (lanes 9 and 10).** The odd-number lanes are for the negative controls with memory tags only and without input gDNA, and the even numbers are for the positive controls with both memory tags and input gDNA. The learning products were analyzed using the ethidium bromide staining 4-20% Urea-PAGE at 60°C. The contrasts of the top image for LP_H_ and the bottom image for LP_s_ were adjusted separately from the orginal Urea-PAGE image (see Additional file 2: Figure S2) to better assess the results.

To further minimize the undesirable LP_M-M_ and maximize the yield and efficiency of the learning protocol to capture and store the information from inputs, the learning protocol was conducted at various annealing temperatures and the amount of extended strands was evaluated as compared to the negative control with memory tags only (Figure 4; see Additional file 2: Figure S2). Comparing LP_H_ on the bases of the gel intensity analyses, only for the positive control with memory tags and input DNA (Figure 4; see Additional file 2: Figure S2, even number lanes), significantly more extended strands were present at the annealing temperatures of 40°C (2.4 fold and 2.1 fold, respectively) and 55°C (2.3 fold and 2 fold, respectively) than those at 25°C and >60°C. No LP_H_ was observed for the negative control. This can be attributed to not only the increased specificity of memory tags to inputs at those relatively high temperatures (as compared to lower temperatures, *e.g.*, 25°C), but also more favorable annealing conditions between memory tags and inputs (as compared to higher temperatures, *e.g.,* >60°C). Comparing LP_S_, its concentration decreased as temperature elevated due to the increase of melting temperature. Particularly, there was significantly less LP_S_ at 55° (0.72 fold and 0.65 fold, respectively) and 60°C (0.48 fold and 0.42 fold, respectively) than those at 25° and 40°C, and no significant differences between positive and negative controls at >55°C. This implies that LP_S_ would contain much less undesirable LP_M-M_ (*i.e.,* purer) at the higher temperatures. However, at the temperature over 60°C, none to very little LP_S_ was yielded. These results suggest that the annealing temperature of 55°C could provide an optimal condition to maximize LP_I-M_ and minimize LP_M-M_. For the rest of the study, 55°C was used as the annealing temperature.

#### Associative recall with microarray: proof of principle

The capabilities of the associative recall procedure were tested using a microarray detection platform with the learned products from the two model bacterial strains, *i.e., E. coli* and *B. subtilis*, which, according to our preliminary analysis, were shown to be genomically very different (see Additional file 3: Table S1). For example, less than 6% of 14-base sequences were common in the digested gDNA of both strains. Therefore, the number of common sequences at the length of the learned products (*i.e.,* >50 bases) must be very low between two bacterial strains.

**Figure 5:**
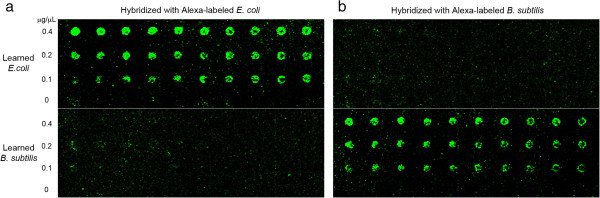
**Microarray-based Memory protocol.** Microarray image analyses of the learned products from *E. coli* and *B. subtilis* gDNA after hybridization with **(a)** Alexa-labeled *E. coli* gDNA and **(b)** Alexa-labeled *B. subtilis* gDNA. The microarray slide was scanned with GenePix 4000B at 100% of laser power with a PMTG setting of 1,000 (see Additional file 4: Table S2).

**Figure 6:**
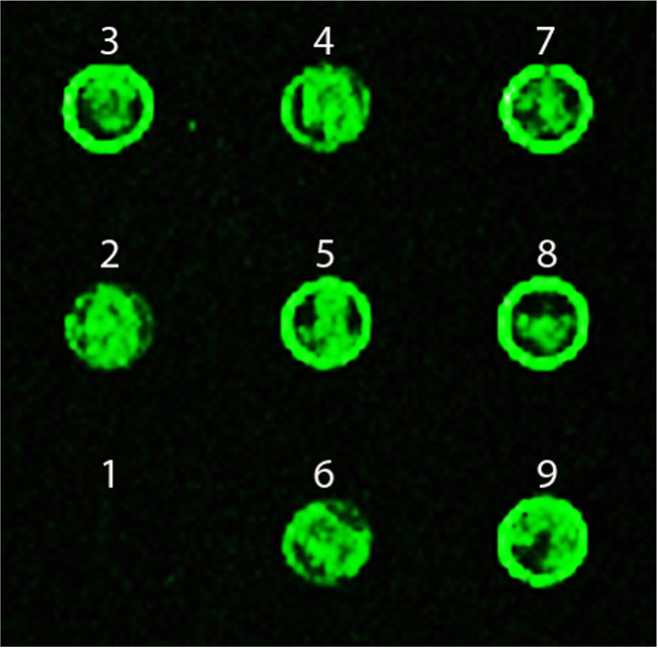
**Sensitivity of the microarray-based Memory protocol.** Samples with different concentrations of *E. coli* gDNA (1, 0%; 2, 10%; 3, 20%;4, 30%; 5, 40%, 6, 50%, 7, 60%, 8, 80% and 9, 100%) and same order of the rest with gDNA from *B. subtilis* were learned and spotted on the CodeLink® microarray slide. Recall was done through the hybridization with Alexa-labeled *E. coli* gDNA. The microarray slide was scanned at 100% of laser power with a PMTG setting of 1,000.

Three different concentrations of the learned products were printed onto microarray slide with 10 replications each, and the Alexa-labeled *E. coli* or *B. subtilis* gDNA were hybridized as probes. Figure 5 shows the scanned images of the microarray slide after each hybridization. Visually comparing the same features in two different hybridizations and different features in the same hybridization, Alexa-labeled *E. coli* probes resulted in significantly higher intensity for all the *E. coli* spots than *B. subtilis* spots, and Alexa-labeled *B. subtilis* probes yielded higher spot intensity for all the *B. subtilis* spots. This clearly demonstrates the pattern separation capability of the Biological Memory that can discriminate differences in inputs and classify the identity of the sample, verifying the basic operation of the Biological Memory system. To further evaluate its sensitivity, the Memory protocol was implemented using mixtures of gDNA from the two different bacterial strains. Samples with different concentrations of *E. coli* gDNA and equal parts of the rest with gDNA from *B. subtilis* were learned and spotted on the microarray slide. After recall with Cy3 dye labeled *E. coli* gDNA through hybridization, all the spots with learned *E. coli* gDNA lighted up, except the spot without *E. coli* gDNA (Figure 6). Even with only 10% of *E. coli* gDNA in the background of *B. subtilis*, it can still be learned and recalled. Therefore, the learning and recall protocols were able to detect the target at very low concentration in the mixture of different strains, indicating its capability of very fine level of resolution to learn the inputs, recall similar DNAs, differentiate between dissimilar DNAs, and detect small concentration differences in samples, both pure and mixed in different degrees.

To analyze the signal intensity difference, BSI and signal-to-noise ratio (SNR) values were extracted using GenePix® Pro 6.0 at three different PMTG values as sensitivity settings, *i.e*., 800, 900 and 1000. However, the microarray image analyses showed that the signal intensity of spots could not be compared and analyzed statistically, because of the low SNR values of features. To obtain a reliable signal intensity value, for example, to estimate a limit of detection (LOD) and a limit of quantification (LOQ), the SNR value should be higher than 3 [17, 18]. However, even though they could be visually discriminated as described above, most of features showed SNR values lower than 3 (see Additional file 4: Table S2). The low SNR, *i.e.*, low signal intensity, of each spot could be related to the probe length and/or the low discrimination power of the microarray [17]. More uniformly digested and shorter DNA would allow increasing the efficiency of the probe-target hybridization on the microarray. The discrimination power by microarray should be also further enhanced. Increasing the number of fluorescent dyes per probe strand would be one option to increase the signal intensity. Exploring other higher resolution detection system for the recall protocol would be another option, such as the Luminex® system, which is a fluorescent bead-based multiplex assay system for the quantitation and detection of biomolecules, including DNA.

Furthermore, to realize the potential of the Memory protocol that was demonstrated in this study, the protocol should be further generalized to validate its ability to distinguish among different species and strains, and their levels and patterns of gene expression. A microarray could be generated that has spots corresponding to the learned memory strands of gDNAs or cDNAs from different species, strains, or combinations of organisms. Each spot represents the learned product of an organism or a collection of organisms. Unknown biological samples (*e.g.,* gDNAs or cDNAs from real-world samples such as soil, water, and biological specimens) are learned and their learned memory products are recalled with the gDNA or cDNA microarray. Based upon the differences in the hybridization patterns of the learned products, inferences could be made as to the genomic contents and states of the unknown biological samples without their explicit genomic information. Moreover, the technical platform of the recall protocol could be further refined to make the Biological Memory more practical by using oligonucleotide arrays with specific oligonucleotides in known locations on the chip. The Affimatrix GeneChip® System (Affimatrix, Inc., Santa Clara, CA) could produce an oligonucleotide chip with ~9 × 10^5^ different oligonucleotide spots, each of which contains millions of copies of a specific oligonucleotide with a specific length, via the company’s proprietary light directed chemical synthesis process. With such oligonucleotide arrays, hybridization signatures of the learned memory products of unknown biological samples (*e.g.,* gDNA or cDNA from the environment or a living being) could be captured and classified. Comparative analyses of the hybridization patterns and signal intensities of the learned memory products at different time intervals or locations could allow us to effectively sense changes in the genomic state of a biological system of interest and make inferences about the biosystems without their clear genomic information, realizing the promise of the Biological Memory. An added benefit of the oligonucleotide arrays would be that we could acquire sequence information on the learned products on the basis of the known sequence information of oligonucleotides on the array, further expanding the utility of the Biological Memory. Hence, with further improvements as the technology progresses, the Biological Memory protocol may catalyze a paradigm shift in biosensing, from the biomonitorings of ecosystems to the diagnoses of diseases, by allowing the assessment of the large amounts of genetic information or all expressed genes in *in situ* biological samples at the population or community scale.

## Conclusions

This study developed and verified an *in vitro* Biological Memory to capture and store genomic information from biological samples, both known and unknown, and to classify and compare their genomic patterns. By processing genomic information *in vitro*, rather than *in silico*, the advantages include massively parallel sampling of the input DNA, ability to work with unknown organisms and sequences, and massively parallel recall and matching of DNA sequence content to detect changes and classify them. Experimental results with two model bacterial strains demonstrated that the protocols worked as designed, and were able to resolve small differences in sequence content. Specifically, the developed learning protocol was simple, fast, and flexible and could effectively capture the genomic contents of samples without their explicit genomic information at the population or community scale. Also, the recall protocol could discriminate genomic patterns with very fine level of resolutions by template matching reactions between the learned memory molecules and new inputs using the microarray detection platform. However, further improvements are required to fully realize its potential. The learning protocol should be further optimized and generalized to increase the learning capacity, and the discrimination power of the recall protocol by microarray should be further improved. Particularly, it should be not only validated using more complex genomic samples, but also tested and optimized for gene expression studies (*i.e.,* using cDNA). With further improvements, optimizations, and generalizations, it is expected that not only the excellent promise of the Biological Memory system could be realized as the genomic pattern classifier, but also its applicability could be expanded to other various biological and biomedical as well as computational applications. The key point is that biological information is processed without explicit knowledge of its sequence content in order to detect relative changes between samples. Examples include an environmental monitoring method that could provide a holistic view of the genomic status of an ecosystem, a screening tool for the prognosis of human diseases, such as cancer and bacterial or viral infectious disease, a biosensing platform for pathogens in water and food sources, and large-scale DNA-based memories for massive storage and retrieval of non-biological information.

## Methods

### DNA samples

The initial memory oligonucleotide with chemically modified 5′ end, 20-base NCH tag sequence, and 20-base random sequence was designed and purchased from IDT DNA technology Inc. (Coralville, IA): 5′-/Bio (or Am)/GAA AAA ACA CCC CTT CGA TGN NNN NNN NNN NNN NNN NNN N-3′ (mole. wt. 12,668.4 g/mole). To ensure its purity and full randomization, it was purchased with the options of high pressure liquid chromatography (HPLC) purification and hand-mixing. The NCH tag sequence was carefully designed to minimize undesirable cross-hybridization and experimentally confirmed by the previously established methods [10, 13].

The gDNA of *E. coli* K12 and *B. subtilis* was extracted by the phenol extraction and ethanol precipitation methods described elsewhere [19]. The extracted gDNA was further digested enzymatically to fragments with an average length of around 200 bases to minimize the formation of secondary structures by the long gDNA and maximize the efficiency of the learning protocol in Biological Memory. The gDNA digestion was implemented according to the previously established method [19] with some modifications using deoxyribonuclease I (DNase I), a random endonuclease, which produces single-strand nicks in the presence of Mg^2+^, randomly cleaving each strand of double strand DNA. Briefly, the reaction mixture contained gDNA (0.2 μg/μL), and pancreatic DNase I (Amersham Biosciences, Piscataway, NJ) (0.002 U/μL) in 10 mM Tris–HCl (pH 7.5) and 25 mM MgCl_2_. After incubation at 37°C for 90 sec, the reaction was terminated by adding EDTA (5 mM). The digested gDNA was purified by the phenol extraction and ethanol precipitation. The purified gDNA was evaluated by the denatured Urea polyacrylamide gel analyses (8 M Urea, 4% stacking gel, 12% resolving gel, 1× Tris-Borate-EDTA buffer) at 60°C. Also, the concentration of the digested gDNA was evaluated using DU® 800 Ultraviolet/Visible/Near-Infrared spectrophotometer (Beckman Coulter, Brea, CA) at the wavelength of 260 nm.

### *In vitro*learning protocol

The reaction mixture consists of 0.02 μg/μL of the digested gDNA (*i.e.,* the input DNA), and 1.6 μM of 5′-amine or biotin modified memory tag in TAEMg buffer (10 mM Tris-accetate, 1 mM EDTA, 10 mM Mg-accetate) as a final concentration. The mixture was incubated at 95°C for 5 min. After a brief centrifugation, it was gradually cooled down to a designated temperature (typically 25° or 55°C) for 30 min, followed by 30-min incubation at 37°C after adding Klenow fragment (5 U/μL) and dNTPs mixture (10 mM). *E. coli* exonuclease I (*Exo* I) (20 U/μL) was added, followed by additional incubation for 30 min. During the optimization of the *in vitro* learning protocol, the initial memory tags with biotin modified 5′ end was used and the learned products were purified using Dynabeads® M-270 streptavidin (Invitrogen corporation, Carlsbad, CA) according to the manufacturer’s instruction. The purified learned products were evaluated by the denatured Urea-PAGE analyses (8 M Urea, 4% stacking gel, 20% resolving gel, 1× Tris-Borate-EDTA buffer) at 60°C. The band area intensity of the Urea-PAGE image was assessed on the basis of the gel intensity analysis with ImageJ software (http://imagej.nih.gov) [20]. For the microarray-based recall, the learning protocol was implemented using the memory tags with amine modified 5′ end.

### Associative recall protocol with microarray

The learned products with 5′-amine modified memory tags was purified by the phenol extraction and ethanol precipitation, resuspened in 1× microarray printing buffer (50 mM sodium phosphate [pH 8.5]) as a stock solution (1 μg/μL), and serially diluted for printing with the printing buffer. The 30 μL of the learned products, a negative control (*i.e.,* learned products without input DNA), and a blank (*i.e.,* 1× printing buffer only) were transferred into a 384-well plate. Each sample in the well plate was printed with 10 replications onto CodeLink™ activated microarray slide (Amersham Biosciences) using MicroGrid II microarray printing system with BioRobotics MicroSpot 2500 pins (Genomic Solutions, MI) at 40% relative humidity according to the previously established protocol [17]. After printing, the slides were stored in a customized hybridization chamber (GeneTix, CA), which was filled with saturated NaCl solution at bottom, for 15 hr at ~75% of relative humidity to couple the amine group at the 5′ end of the learned products to the activated N-hydroxysuccinimide-ester group on the microarray slide surface. For blocking, the slide was kept in a pre-warmed blocking solution (0.1 M Tris, 50 mM ethanolamine [pH 9.0]) at 50°C for 30 min. The slide was briefly rinsed twice with deionized water and washed with pre-warmed 4× saline-sodium citrate (SSC) buffer with 0.1% sodium dodecyl sulfate (SDS) for 30 min at 50°C with gentle shaking. The slide was dried by centrifugation and stored at ambient temperature. To evaluate the recall protocol, each digested *E. coli* and *B. subtilis* gDNA was labeled using the ULYSIS™ Alexa Fluor 532® Nucleic Acid Labeling Kit, according to the manufacturer’s instruction (Invitrogen corporation, Carlsbad, CA). The ULYSIS™ Nucleic Acid Labeling allows a fluorescent dye to react with the N_7_ of a purine base (*i.e.,* A or G) in a nucleic acid to form a stable coordination complex, implying the high possibility of labeling the entire strands of the digested gDNA. After labeling, the Alexa-labeled *E. coli* or *B. subtilis* gDNA was stored in 4× SSC with 0.1% SDS. For the recall, the labeled target DNA was reconstituted in the hybridization solution (7.5× SSC, 37.5% formamide, 0.15% SDS, 0.3 μg/μL bovine serum albumin) as a final concentration of 0.025 μg/μL. The 20 μL of the target DNA solution was applied to the printed microarray slide with LifterSlip™ microarray cover slide. Hybridization was performed at room temperature for 20 hr in the customized hybridization chamber with ~75% relative humidity. After hybridization, it was washed at room temperature twice with 4× SSC containing 0.2% SDS for 5 min each, 1× SSC for 10 min, and twice with 0.2× SSC for 2 min each. Microarray slide was completely dried by centrifugation.

### Image acquisition and data analysis

After recall, the microarray slide was scanned with GenePix 4000B (Axon Instruments, Forester City, CA) at 100% of laser power with 3 different PMTG settings from 800, 900, and 1,000 as detection sensitivity settings. Photobleaching was very minimal at the scanner settings. Signal intensities were measured with GenePix® Pro 6.0 microarray image analysis software (Axon Instruments, Forester City, CA) with 10 μm of pixel size as a detection sensitivity. The obtained data were saved as csv (comma delimited) format for data analysis. Background-subtracted intensity (BSI) values were used in the subsequent analyses. Statistical analyses were performed using statistical package R (version 2.8.0) [21].

## Electronic supplementary material

Additional file 1: Figure S1: Estimation of the by-products by the undesirable annealing and extension between memory tags themselves during the learning protocol. (PDF 126 KB)

Additional file 2: Figure S2: The original Urea-PAGE analyses of learning at various annealing temperatures for the protocol optimization: 25^o^C (lanes 1 and 2), 40^o^C (lanes 3 and 4), 55^o^C (lanes 5 and 6), 60^o^C (lanes 7 and 8), and 70^o^C (lanes 9 and 10). The odd-number lanes are for the negative controls with memory tags only and without input gDNA, and the even numbers are for the positive controls with both memory tags and input gDNA. The learning products were analyzed using the ethidium bromide staining 4-20% Urea-PAGE at 60^o^C. (PDF 390 KB)

Additional file 3: Table S1: Estimated number of common sequences between the gDNA fragments of *E. coli* and *B. Subtilis* at a given length. (PDF 97 KB)

Additional file 4: Table S2: Microarray scanning results of the learned products from (a) *E. coli* gDNA after hybridization with Alexa-labeled *E. coli* gDNA and (b) *B. subtilis* gDNA after hybridization with Alexa-labeled *B. subtilis* gDNA. (PDF 371 KB)
