# Supplementary material for: A DNA-based pattern classifier with in vitro learning and associative recall for genomic characterization and biosensing without explicit sequence knowledge
Source: J Biol Eng. 2014 Nov 6;8:25. doi: 10.1186/1754-1611-8-25 (PMC4237745; doi:10.1186/1754-1611-8-25)
Supplement: Supplementary file 1 — Additional file 1: Figure S1: Estimation of the by-products by the undesirable annealing and extension between memory tags themselves during the learning protocol. (PDF 126 KB) [file 13036_2014_157_MOESM1_ESM.pdf]

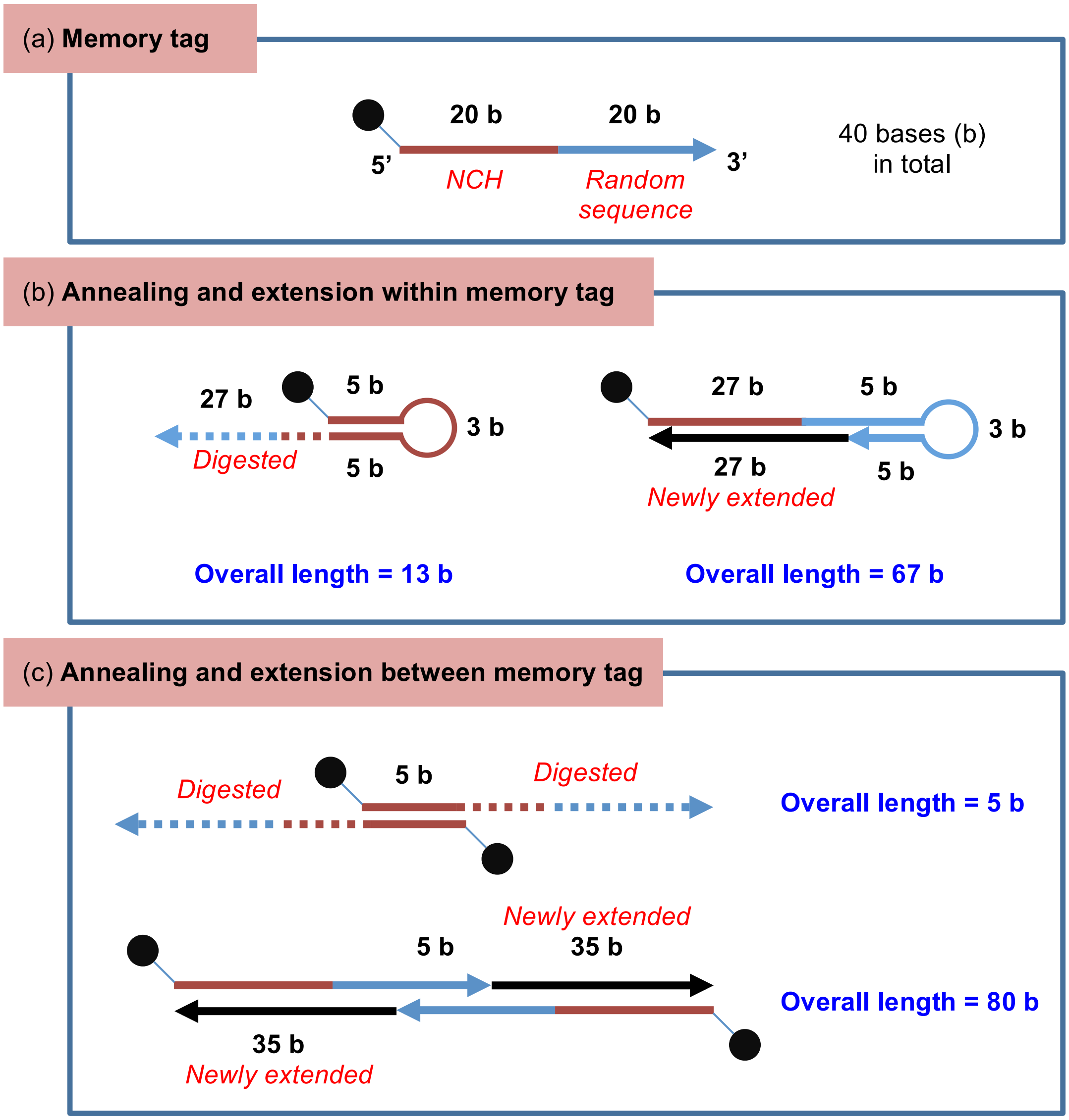

Figure S1 Estimation of the by-products by the undesirable annealing and extension between memory tags themselves during the learning protocol.
