# Supplementary material for: A DNA-based pattern classifier with in vitro learning and associative recall for genomic characterization and biosensing without explicit sequence knowledge
Source: J Biol Eng. 2014 Nov 6;8:25. doi: 10.1186/1754-1611-8-25 (PMC4237745; doi:10.1186/1754-1611-8-25)
Supplement: Supplementary file 2 — Additional file 2: Figure S2: The original Urea-PAGE analyses of learning at various annealing temperatures for the protocol optimization: 25oC (lanes 1 and 2), 40oC (lanes 3 and 4), 55oC (lanes 5 and 6), 60oC (lanes 7 and 8), and 70oC (lanes 9 and 10). The odd-number lanes are for the negative controls with memory tags only and without input gDNA, and the even numbers are for the positive controls with both memory tags and input gDNA. The learning products were analyzed using the ethidium bromide staining 4-20% Urea-PAGE at 60oC. (PDF 390 KB) [file 13036_2014_157_MOESM2_ESM.pdf]

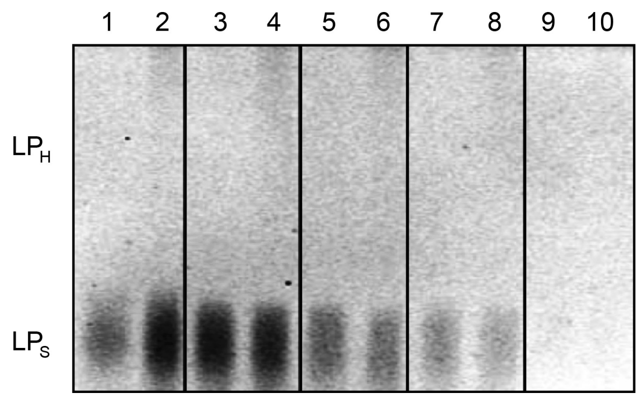

Figure S2 The original Urea-PAGE analyses of learning at various annealing temperatures for the protocol optimization: 25°C (lanes 1 and 2), 40^oC (lanes 3 and 4), 55°C (lanes 5 and 6), 60°C (lanes 7 and 8), and 70°C (lanes 9 and 10). The odd-number lanes are for the negative controls with memory tags only and without input gDNA, and the even numbers are for the positive controls with both memory tags and input gDNA. The learning products were analyzed using the ethidium bromide staining 4-20% Urea-PAGE at 60°C.
