# Supplementary material for: A DNA-based pattern classifier with in vitro learning and associative recall for genomic characterization and biosensing without explicit sequence knowledge
Source: J Biol Eng. 2014 Nov 6;8:25. doi: 10.1186/1754-1611-8-25 (PMC4237745; doi:10.1186/1754-1611-8-25)
Supplement: Supplementary file 3 — Additional file 3: Table S1: Estimated number of common sequences between the gDNA fragments of E. coli and B. Subtilis at a given length. (PDF 97 KB) [file 13036_2014_157_MOESM3_ESM.pdf]

Computational estimation of common sequences between the genomic DNA fragments of Escherichia coli and Bacillus subtilis

The genomic DNA (gDNA) sequences of *E. coli* and *B. subtilis* were obtained from the homepage of National Center for Biotechnology Information (NCBI) (http://www.ncbi.nlm.nih.gov/) as FASTA format. A computation program, which was written by the Practical Extraction and Report Language (PERL), was used not only to extract unique sequences at a given length from the genomic information of each strain, but also to compare them and estimate the number of common sequences. Table S1 shows the number and percent of common sequences between *E. coli* and *B. subtilis* at a given length in the range of 10 – 14 bases. The computational analyses for longer sequences could not be performed due to the high computational costs as the sequence lengths increase. However, our preliminary estimation indicates that less than 6% of 14-base sequences are common in the digested gDNA of both strains. This implies that the number of common sequences at the length of the learned products (*i.e.,* >50 bases) must be very low between two bacterial strains.

Table S1 Estimated number of common sequences between the gDNA fragments of *E. coli* and *B. Subtilis* at a given length

Sequence length (bases)	EC ∩ BS [1]		BS ∩ EC [2]	
	Number of common sequences	%	Number of common sequences	%
10	9.044×10^6	97.5	8.234×10^6	97.7
11	7.348×10^6	79.2	6.941×10^6	82.4
12	4.087×10^6	44.0	4.043×10^6	48.0
13	1.596×10^6	17.2	1.602×10^6	19.0
14	0.508×10^6	5.5	0.509×10^6	6.0

[1] Sequences of *E. coli* gDNA fragments at a given length that are present in the *B. subtilis* genome
[2] Sequences of *B. subtilis* gDNA fragments at a given length that are present in the *E. coli* genome
The estimated numbers of common sequences are different because there are duplicated sequences in the gDNA fragments of each strain at a given length.
