# Supplementary material for: A DNA-based pattern classifier with in vitro learning and associative recall for genomic characterization and biosensing without explicit sequence knowledge
Source: J Biol Eng. 2014 Nov 6;8:25. doi: 10.1186/1754-1611-8-25 (PMC4237745; doi:10.1186/1754-1611-8-25)
Supplement: Supplementary file 4 — Additional file 4: Table S2: Microarray scanning results of the learned products from (a) E. coli gDNA after hybridization with Alexa-labeled E. coli gDNA and (b) B. subtilis gDNA after hybridization with Alexa-labeled B. subtilis gDNA. (PDF 371 KB) [file 13036_2014_157_MOESM4_ESM.pdf]

Table S2 Microarray scanning results of the learned products from (a) *E. coli* gDNA after hybridization with Alexa-labeled *E. coli* gDNA and (b) *B. subtilis* gDNA after hybridization with Alexa-labeled *B. subtilis* gDNA

(a)

**$[LP]_{EC}$ (µg/µL)**	**F532 Median**	**F532 Mean**	**F532 SD**	**B532 Median**	**B532 Mean**	**B532 SD**	**BSI**	**F532 Total Intensity**	**SNR**
0.4	3,195	3,384	1,421.2	1,888	2,042	964.8	1,342	270,717	1.39
0.2	2,704	2,749	1,066.8	1,842	1,915	795.4	834	219,944	1.05
0.1	2,389	2,476	959.6	1,775	1,838	706.8	638	198,044	0.90
0	1,776	1,932	855.7	1,709	1,768	706.5	164	154,576	0.23

(b)

**$[LP]_{BS}$ (µg/µL)**	**F532 Median**	**F532 Mean**	**F532 SD**	**B532 Median**	**B532 Mean**	**B532 SD**	**BSI**	**F532 Total Intensity**	**SNR**
0.4	2,731	2,836	1,223.8	1,732	1,788	726.4	1,048	226,877	1.44
0.2	2,331	2,405	995.3	1,730	1,778	688.8	627	192,425	0.91
0.1	2,144	2,240	884.2	1,747	1,791	707.8	449	179,214	0.63
0	1,741	1,953	1502.0	1,678	1,728	688.6	225	156,250	0.33

The title of each column represents:

- $[LP]_{EC}$ = Concentration of the learned product from *E. coli* gDNA on a microarray spot
- $[LP]_{BS}$ = Concentration of the learned product from *B. subtilis* gDNA on a microarray spot
- F532 Median = Median Alexa 532® intensity
- F532 Mean = Mean Alexa 532® intensity
- F532 SD = Standard deviation of the Alexa 532® intensity
- B532 Median = Median Alexa 532® background intensity
- B532 Mean = Mean Alexa 532® background intensity
- B532 SD = Standard deviation of the Alexa 532® background intensity
- BSI = Background subtracted intensity (*i.e.,* F532 Mean – B532 Mean)
- F532 Total Intensity = Total Alexa 532® intensity
- SNR = Signal to noise ratio (*i.e.*, BSI/[B532 SD])

Each intensity value in Table S2a and S2b represents an average intensity of 10 spots at a concentration of the learned product as shown in Figure 5a, top and 5b, bottom, respectively.

The microarray slide was scanned with GenePix 4000B at 100% of laser power with a PMTG setting of 1,000 and analyzed with a diameter of 100 µm.
